# Evaluating the monophyly of *Mammillaria* series *Supertextae* (Cactaceae)

**DOI:** 10.3897/phytokeys.177.62915

**Published:** 2021-04-28

**Authors:** Cristian R. Cervantes, Silvia Hinojosa-Alvarez, Ana Wegier, Ulises Rosas, Salvador Arias

**Affiliations:** 1 Jardín Botánico, Instituto de Biología, Universidad Nacional Autónoma de México, Tercer Circuito Exterior, Ciudad Universitaria, Coyoacán, Ciudad de México 04510, Mexico; 2 Posgrado en Ciencias Biológicas, Instituto de Biología, Universidad Nacional Autónoma de México, Ciudad Universitaria, Coyoacán, Ciudad de México, 04510, Mexico; 3 Tecnologico de Monterrey, School of Engineering and Sciences, Ave. Eugenio Garza Sada 2501, Monterrey, N.L. 64849, Mexico

**Keywords:** Bayesian inference, Cactaceae, chloroplast DNA, *Mammillaria haageana*, molecular phylogeny, *M.* ser. *Supertextae*, taxonomy

## Abstract

*Mammillaria* (Cactaceae) taxonomy has been historically problematic due to the morphological variability and sympatry of the species. This has led to several proposals for infrageneric classification, including subgeneric, section and series categories. Mammillaria ser. Supertextae is one of 15 series and is made up of a variable set of species that are mainly distributed in southern Mexico and Central America. However, the phylogenetic relationships within M. ser. Supertextae and its relationship to other *Mammillaria* taxa are far from fully understood. Here we attempt to elucidate these relationships using complete terminal sampling and newly obtained chloroplast marker sequences and comparing them to *Mammillaria* species sequences from GenBank. Our phylogenetic analyses showed that M. ser. Supertextae comprises a well-supported monophyletic group that diverged approximately 2.1 Mya and has M. ser. Polyacanthae as its sister group; however, relationships within M. ser. Supertextae remain unresolved. The topology obtained within M. ser. Supertextae must also be interpreted under the distribution shared by these taxa, but it is difficult to differentiate ancestral polymorphisms from possible introgression, given the short time elapsed and the markers used. Our results show that the infrageneric units of *M. haageana* and *M. albilanata* can be considered independent evolutionary units. We also suggest that the relationship between *M. haageana* and *M. albilanata* is convoluted because their distribution overlaps (mainly towards southern Mexico), with genetic differences that possibly indicate they represent more than two taxonomic entities. One possible explanation is that there could still be gene flow between these taxa, and we might be witnessing an ongoing speciation process.

## Introduction

*Mammillaria* Haw. (Cactaceae, Cactoideae, Cacteae) is the most diverse genus within the cactus family, with a broad range of recognized species, ranging from 163 (Hunt et al. 2006) to 181 (Pilbeam 1999) up to 320 species (Reppenhagen 1992). *Mammillaria* is characterized by tuberculate stems, definite dimorphic areoles not connected by a groove, flowers that arise from the base of the tubercles and not apically, and seeds with testa cell walls that are par-concave and undulated (Bravo-Hollis and Sánchez-Mejorada 1991; Lüthy 1995; Anderson 2001; Hunt et al. 2006). This genus, together with *Coryphantha* (Engelm.) Lem., *Escobaria* Britton & Rose, *Neolloydia* Britton & Rose, and *Ortegocactus* Alexander, is integrated into the Mammilloid clade (Butterworth et al. 2002). However, it has been proposed that *Mammillaria* is a polyphyletic group. Breslin et al. (2021), using plastid genomes, confirmed previous studies showing *Mammillaria* is nonmonophyletic, as currently circumscribed, so they proposed that the Mammilloid clade be circumscribed in three monophyletic genera, *Mammillaria* s.s., *Coryphantha* and *Cochemiea* s.l., as previously suggested by Vázquez-Sánchez et al. (2013). Furthermore, the taxonomy of *Mammillaria* has historically been difficult due to large morphological variability, phenotypic plasticity, sympatric distribution of species, and suspected hybridization events.

Within *Mammillaria*, there are 15 recognized series (Hunt et al. 2006), one of which is M. ser. Supertextae D.R. Hunt, distributed from western Mexico to Central America and even the Caribbean islands (Pilbeam 1999). Mammillaria ser. Supertextae is a clear example of the species delimitation problem within the genus, illustrated by the number of accepted species ranging from 8 to 27, although the most recent taxonomic proposal recognizes only 9 taxa (Table 1). Morphometric and molecular studies have attempted to assess the proposed infrageneric relationships for *Mammillaria*, but none have been specifically directed at M. ser. Supertextae. Lüthy (1995) performed a detailed morphological analysis of the genus with a phenetic approach, in which he included four species of M. ser. Supertextae (*M. albilanata* Backeb., *M. dixanthocentron* Backeb. ex Mottram, *M. haageana* Pfeiff., and *M. huitzilopochtli* D.R. Hunt), showing that M. ser. Supertextae is characterized by the presence of extracellular crystals; however, the trait is not exclusive to M. ser. Supertextae, as the M. ser. Leucocephalae Lem. ex Schumann also shows this characteristic. Butterworth and Wallace (2004) conducted a molecular study using two chloroplast markers (*rpl*16 and *psb*A-*trn*H), including five species of M. ser. Supertextae (same as Lüthy but including *M. supertexta* Mart. ex Pfeiff.); although the species were grouped together, the support values were low (BS = 63, PP = 0.99), and a phylogenetic relationship could not be established with the remainder of *Mammillaria*. More recently, the complete sequencing of the chloroplast genome of eight *Mammillaria* species confirms that four M. ser. Supertextae taxa (*M. crucigera* Mart., *M. supertexta*, *M. huitzilopochtli*, M. haageana subsp. san-angelensis (Sánchez-Mej.) D.R. Hunt) represent a clade (Solórzano et al. 2019; Hinojosa-Alvarez et al. 2020).

**Table 1.** Historical account of taxonomic classifications of M. ser. Supertextae D.R. Hunt (= *Elegantes*).

Backeberg (1961)	Bravo-Hollis and Sánchez-Mejorada (1991)	Reppenhagen (1992)	Lüthy (1995)	Hunt et al. (2006)
*M. crucigera* Mart.	*M. huitzilopochtli* D.R.Hunt	*M. elegans*	*M. albilanata*	*M. albilanata*
*M. celsiana* Lem.	*M. lanata* Orcutt	*M. meissneri*	*M. columbiana*	*M. crucigera*
*M. elegans* DC.	*M. albilanata* Backeb.	*M. haageana*	*M. eriacantha*	*M. columbiana*
*M. supertexta* Mart. ex Pfeiff.	*M. supertexta*	*M. conspicua*	*M. haageana*	*M. dixanthocentron*
*M. dyckiana* Zucc. ex Pfeiff.	*M. crucigera*	*M. monticola* Repp.	*M. supertexta*	*M. flavicentra*
*M. dealbata* A.Dietr.	*M. dixanthocentron* Backeb.	*M. lanigera* Repp.	*M. crucigera*	*M. haageana*
*M. haageana* Pfeiff.	*M. vaupelii* Tiegel	*M. donatii*	*M. dixanthocentron*	*M. halbingeri*
*M. acanthoplegma* Lehm.	*M. haageana*	*M. albidula* Backeb.	*M. huitzilopochtli*	*M. huitzilopochtli*
*M. meissneri* Ehrenbg.	*M. collina* J.A.Purpus	*M. lanata*		*M. supertexta*
	*M. donatii* Berge ex K.Schum.	*M. tlalocii* Repp.		
	*M. san-angelensis* Sánchez-Mej.	*M. huitzilopochtli*		
	*M. martinezii* Backeb.	*M. crucigera*		
	*M. fauxiana* Backeb.	*M. flavicentra*		
	*M. conspicua* J.A.Purpus	*M. dixanthocentron*		
	*M. halbingeri* Boed.	*M. supertexta*		
	*M. flavicentra* Backeb.	*M. reppenhagenii*		
	*M. tegelbergiana* G.E.Linds.	*M. albilanata*		
	*M. reppenhagenii* D.R.Hunt	*M. igualensis* Repp.		
	*M. ruestii* Quehl	*M. tegelbergiana*		
	*M. yucatanensis* Orcutt	*M. igniota* Repp.		
		*M. halbingeri*		
		*M. noureddineana* Repp.		
		*M. columbiana* Salm-Dyck		
		*M. ruestii*		
		*M. yucatanensis*		
		*M. chilapensis* Repp.		
		*M. eriacantha* Link & Otto ex Pfeiff.		

To disentangle the evolution of *Mammillaria*, we decided to focus on elucidating the phylogenetic relationships of M. ser. Supertextae. We included all taxa proposed by Hunt et al. (2006), except for *M. halbingeri* Boed., as, according to Reppenhagen (1992), the species was not reported again. We also included 12 localities of *M. haageana* and seven of *M. albilanata*. All these species together constitute one taxonomic complex within M. ser. Supertextae (Arias et al. 2012). We chose two chloroplast markers, the *rpl*16 intron and the intergenic spacer *psb*A-*trn*H. In Cactaceae, both markers have been used to resolve phylogenetic relationships (Korotkova et al. 2010; Sánchez et al. 2014; Cruz et al. 2016; Barrios et al. 2020), including *Mammillaria* (Butterworth and Wallace 2004; Vázquez-Sánchez et al. 2013; Hernández‐Hernández et al. 2014); therefore, there are many sequences available in GenBank that can be used to expand the sampling of terminals, including sister groups and outgroups essential for testing the monophyly (Korotkova et al. 2017) of M. ser. Supertextae. The main objective of this study was to test the monophyly of M. ser. Supertextae and estimate its divergence time by broadening the sample of terminals within the series.

## Materials and methods

The present study included a total of 123 taxa, 111 species of *Mammillaria*, 5 closely related genera (*Escobaria*, *Pelecyphora* Ehrenb., *Coryphantha*, *Neolloydia*, and *Ortegocactus*) and three external groups (*Ferocactus haematacanthus* (Salm-Dyck) Borg ex Backeb., *Ferocactus latispinus* (Haw.) Britton & Rose, and *Stenocactus lloydii* Berger). We selected two chloroplast loci: the intron *rpl*16 and the intergenic spacer region *psb*A-*trn*H. We downloaded 95 sequences of the genus *Mammillaria* (Butterworth and Wallace 2004; Hernández‐Hernández et al. 2011; Fehlberg et al. 2013) from GenBank (see Appendix 1). For M. ser. Supertextae, we obtained two sequences: M. albilanata subsp. tegelbergiana (H. E. Gates ex G. E. Linds.) D.R. Hunt and M. columbiana Salm-Dyck subsp. columbiana from Vázquez-Sánchez et al. (2013). For the remaining 28 taxa of M. ser. Supertextae, we generated new sequence data.

DNA was extracted from 40 mg of silica-dried (24 h) stems. The samples were stored at -80 °C, and 12 hours later, they were triturated in a TissueLyser II (Qiagen, Venlo, Netherlands) at 29 rpm for 25 s twice. Extraction was performed with the DNeasy Plant Mini Kit (Qiagen, Hilden, Germany) following the manufacturer’s instructions, and the elution volume was 35 µl twice in Milli-Q water. The *rpl*16 intron and *psb*A-*trn*H intergenic spacer were amplified using standard PCR protocols. The *rpl*16 region was amplified using the primers from Hernández‐Hernández et al. (2011), *rpl*161F (5ʹ‐GCTATGCTTAGTGTGTGACTCGTT‐3ʹ) and *rpl*163R (5ʹ‐CTTCTATTTGTCTAGGCGTGATCC‐3ʹ), by initially denaturing the DNA for 5 min at 94 °C, followed by 28 cycles of 1 min at 94 °C, 50 s at 55 °C, and 2 min at 72 °C, and a final extension of 4 min at 72 °C. The *psb*A-*trn*H intergenic spacer was amplified with primers from Korotkova et al. (2010), *CApsbA* (5ʹ‐CCGTGCTAACCTTGGTATGG‐3ʹ) and *CAtrnH* (5ʹ‐CCGCGAATGGTGGATTCACAAT‐3ʹ). PCR conditions were 2 min at 94 °C; followed by 29 cycles of 30 s at 94 °C, 30 s at 52 °C; and 1 min at 72 °C; and a final extension of 7 min at 72 °C. Amplifications were performed using 0.6 U of Platinum *Taq* polymerase according to the manufacturer’s protocol (Invitrogen, Carsbad, California, USA), 4 mM mixed dNTPs (Invitrogen, Thermo Fisher Scientific, Waltham, USA), 1.5 mM MgCl2, 16 mg/mL BSA, 0.25 μM each primer, and 30–50 ng of genomic DNA in a reaction volume of 25 μL. The PCR products were sequenced in an Applied Biosystems Sequencer Model 3730xL at the Laboratorio de Biología Molecular de la Biodiversidad y de la Salud, Instituto de Biología, UNAM.

The sequences were aligned in GUIDANCE2 (v. 2.02, Sela et al. 2015) using MAFFT (v. 7.407, Katoh and Standley 2013). The algorithm was implemented with 100 iterations (–msaProgram MAFFT –MSA_Param “\–globalpair \--maxiterate 100” –bootstraps 100). The resulting matrix was imported into PHYDE (v. 0.9971, Müller et al. 2019) to manually edit ambiguously aligned sites. Both genes were concatenated, and data partitions were determined with the program PARTITIONFINDER (v. 2, Lanfear et al. 2017) using the Bayesian information criterion and greedy search. The model with the best fit for both markers was TVM + I + G. Indels and inversion were manually coded with MESQUITE (v 3.6, Maddison and Maddison 2019) using the simple coding method of Simmons and Ochoterena (2000).

Bayesian inference (BI) analysis was performed using MRBAYES (v. 3.2.1, Ronquist et al. 2012). The analysis was run with four Markov chain Monte Carlos (MCMC) (nchains = 4) and ten million generations (ngen = 10000000), sampling trees every 100 generations (samplefreq = 100) and discarding the first 25% as burn-in. All parameters were monitored with TRACER (v. 1.7.1, Rambaut 2018) until they had effective sample sizes (ESS) of greater than 200. Maximum likelihood (ML) analysis was conducted using RAXML (v. 8.2.12, Stamatakis 2014) with molecular and binary partitioning, calculating the conditional likelihood of no invariant data and considering CAT for the heterogeneity of rate. The correction was made with the Lewis (2001) method (-m ASC_MULTICAT –asc-corr=lewis -#1000). To evaluate the monophyly of the subgenera and series recognized by Hunt et al. (2006) in the tree, the Monophy package (v. 1.3, Schwery and O’Meara 2016) of the R program (v. 4.0.3, R Core Team 2018) was used.

To estimate divergence times, we used the credibility interval around the estimated age of the Mammilloid clade (5.83–12.56 Mya; Hernández‐Hernández et al. 2014). We inferred a time-calibrated phylogenetic tree using a BI approach implemented in BEAST (v. 2.6.1, Bouckaert et al. 2019). Analysis of the concatenated matrix used the uncorrelated lognormal relaxed clock (Drummond et al. 2006) for a total of 20 million generations of MCMC, sampling once every 10000 trees and discarding 15% as burn-in using TREEANNOTATOR v. 2.6.0.

## Results

**Figure 1.**
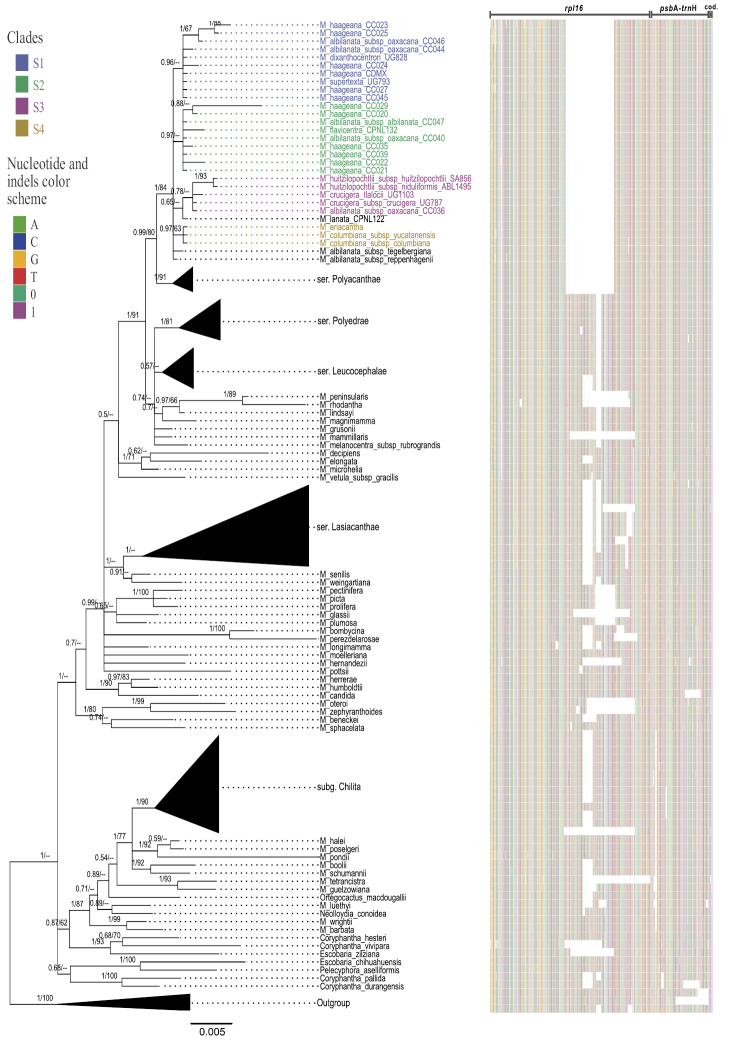
Phylogenetic tree of 123 taxa based on two chloroplast markers using IB. The values on the branches correspond to posterior probability (right) and ML bootstrap (left), and a dash (-) represents values of BS < 60. The left section shows a matrix with variable sites for *rpl*16 and *psb*A-*trn*H, as well as the coding of indels and inversions. Within M. ser. Supertextae, the clades are marked with colors.

The overall sequence matrix for the two genes included 2257 bp and 8 encoded indels. We excluded 1045 bp in *rpl*16 due to uncertain homology. The final length of the aligned matrix for *rpl*16 was 897 bp and 315 bp for *psb*A-*trn*H, with 168 and 69 potentially informative sites, respectively. The BI and ML analyses produced trees with similar topologies (Fig. 1). Mammillaria ser. Supertextae was recovered as a monophyletic group (PP = 1, BS = 84), supported by a transversion in *psb*A-*trn*H. Mammillaria ser. Polyacanthae was also recovered as a monophyletic group (PP = 1, BS = 91), supported by 2 transitions in *psb*A-*trn*H. The sister relationship between M. ser. Supertextae and M. ser. Polyacanthae (PP = 0.99, BS = 80) is supported by a deletion in *rpl*16.

**Figure 2.**
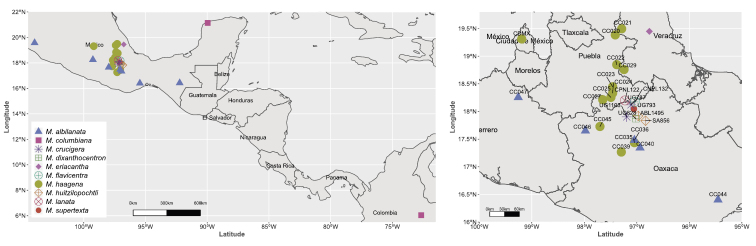
Left distribution of M. ser. Supertextae species sample collection localities; Right inset with southern Mexico shown in greater detail; sampling with emphasis on *M. haageana* and *M. albilanata* are shown, as well as the collection codes (Appendix 1).

A polytomy formed within M. ser. Supertextae, where four clades were formed (S1, S2, S3, and S4), three of which are defined by specific polymorphisms (i.e., clade S1 a transversion in *rpl*16, clade S3 an inversion in *rpl*16). In three clades, at least one terminal of M. albilanata subsp. oaxacana was confirmed (S1: CC044 and CC046; S2: CC040; S3: CC036); in addition, part of its geographic distribution was common to *M. haageana* (Fig. 2A, B). The 12 terminals of *M. haageana* are distributed into two clades. S1 is formed by six terminals of *M. haageana* (CC024, CDMX, CC023, CC025, CC027, and CC045), two terminals of M. albilanata subsp. oaxacana referred to above, *M. dixanthocentron*, and *M. supertexta*, all of which display a transversion in *rpl*16. The second group consists of six terminals of *M. haageana* (CC020, CC021, CC022, CC029, CC035, and CC039), *M. flavicentra*, one terminal of *M. albilanata*subsp. oaxacana, and M. albilanata subsp. albilanata (CC047), all of which had a duplication of 15 bp in *rpl*16. Despite the rather small sampling of polymorphisms, these observations highlight the taxonomic problem in distinguishing *M. haageana* from *M. albilanata*, together with other sister species. The clade S3 formed by M. huitzilopochtli subsp. huitzilopochtli, M. huitzilopochtli subsp. niduliformis, M. crucigera subsp. crucigera, M. crucigera subsp. tlalocii, and one terminal of M. albilanata subsp. oaxacana (CC036) shows an inversion in *rpl*16 of 46 bp, and its sister group was *M. lanata*. Clade S4 is made up of two species, one of which corresponds to *M. columbiana*, which is distributed from Yucatan, Mexico to Colombia and Venezuela (Fig. 2A); the second species is *M. eriacantha*, which is distributed in Veracruz, Mexico (Fig. 2A, B).

**Figure 3.**
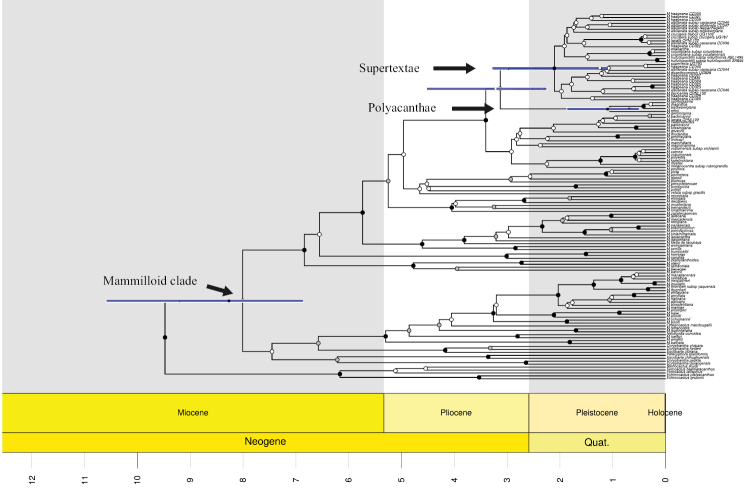
Divergence time estimated using BEAST based on concatenated matrix *rpl*16 and *psb*A-*trn*H. The circles on the nodes represent the PP supports: white < 0.75, gray < 0.95 and black ≥ 0.95. We show the mean divergence times (MDTs) and 95% highest posterior density (HPD) intervals (blue line) to the Mammilloid clade (MDT = 8, HPD = 5.83–11.61), *Supertextae* (MDT = 2.1, HPD = 0.91–3.47), *Polyacanthae* (MDT = 1, HPD = 0.15–2.22), and the sister group relationship (MDT = 2.8, HPD = 1.46–4.73).

The estimated crown age for M. ser. Supertextae was approximately 2.1 Mya (95% HPD = 0.91–3.47) in the Neogene-Quaternary transition (Fig. 3), whereas the M. ser. Polyacanthae crown age was estimated to be approximately 1 Mya (95% HPD = 0.15–2.22) in the mid-Pleistocene (Fig. 3). The divergence between these two groups was approximately 2.8 Mya (95% HPD = 1.46–4.73) in the late Pliocene; however, this clade has low support.

## Discussion

The concatenation of two matrices (*rpl*16 and *psb*A-*trn*H) and extensive sampling (eight of nine species, according to Hunt et al. 2006; Table 1) helped to recover M. ser. Supertextae as a monophyletic group, consistent with previous molecular phylogenetic studies that included only five (Butterworth and Wallace 2004) and three taxa (Solórzano et al. 2019) of the series. The phylogenetic position of *M. eriacantha* has been uncertain, and it was placed within M. ser. Polyacanthae due to the size of its flower (Bravo-Hollis and Sánchez-Mejorada 1991; Hunt et al. 2006). Remarkably, we show that *M. eriacantha* is nested within M. ser. Supertextae, as previously proposed by Reppenhagen (1992) and Lüthy (1995) based on the presence of extracellular crystals. Our results also show that M. ser. Polyacanthae is the sister series of M. ser. Supertextae and that they are part of the Mammillaria sect. Subhydrochylus (Hunt 1987). Within M. ser. Supertextae, phylogenetic relationships were not resolved; however, both *M. haageana* and *M. albilanata* appeared in more than one clade.

Intron *rpl*16 was demonstrated to be the most variable and informative marker compared to the intergenic spacer *psb*A-*trn*H, which is consistent with previous studies in Cactaceae (Korotkova et al. 2010; Vázquez-Sánchez et al. 2013; Cruz et al. 2016). The molecular characteristics that define the monophyly of M. ser. Supertextae and M. ser. Polyacanthae were found in *psb*A-*trn*H, while a deletion in *rpl*16 supports the sister relationship. Although the deletion in *rpl*16 is partial in M. ser. Supertextae and M. ser. Polyacanthae, a total deletion has been reported in members of M. ser. Stylothelae (Butterworth et al. 2007), showing that deletions in *rpl*16 in *Mammillaria* may be a strong characteristic for the identification of infrageneric groups. It remains to be defined whether other polymorphisms, such as the inversion of another chloroplast gene, *trnF*-*GAA*, are also diagnostic of these clades within and between series, as recently reported for *M. crucigera*, *M. huitzilopochtli*, and *M. supertexta* (Solórzano et al. 2019).

The Mammilloid clade originated approximately 8.62 Mya (95% HPD = 5.83–12.56; Hernández-Hernández et al. 2014), and within *Mammillaria*, it is likely that M. ser. Supertextae is a recently divergent group that originated in the Neogene-Quaternary transition approximately 2.1 Mya (95% HPD = 0.91–3.47). In some geographic regions, the M. ser. Supertextae species have undergone tectonic, erosive, alluvial and volcanic changes for millions of years; during the Pleistocene, these processes continued, giving rise to the current geomorphology (Siebert and Carrasco-Núñez 2002; Medina-Sánchez et al. 2020). Paleontological and molecular evidence suggests that glacial climate cycles that occurred during the last 2.5 Mya affected the distribution, diversity, and genetic structure of plant and animal populations (Gámez et al. 2014; Scheinvar et al. 2016; Cornejo-Romero et al. 2017). The hypotheses suggest that during the Pleistocene, these species sought refuge during adverse environmental conditions and expanded again when conditions improved (Scheinvar et al. 2016). Our results show that within M. ser. Supertextae, four clades are formed, two of which have distinctive climatic and topographic characteristics: Clade S1 (*M. haageana*, *M. albialanta*, *M. dixanthocentron* and *M. supertexta*), with species that are distributed in warm zones mainly at altitudes ranging from 447 to 2318 meters in thorn and tropical deciduous forests; and Clade S2 (*M. haageana*, *M. albilanata* and *M. flavicentra*), with species that are distributed in temperate zones at altitudes that range mainly from 1285 to 2518 meters in pine-oak forests. The environmental, geological and topographic differences between closely related species produced during climatic changes suggest differential selection pressures and local adaptation, which could have driven the speciation process (Mastretta‐Yanes et al. 2015; Aquino et al. 2021), as has been suggested for *Mammillaria pectinifera* (Cornejo-Romero et al. 2014), *Cephalocereus columna-trajani* (Cornejo-Romero et al. 2017) and the genus *Epithelantha* (Aquino et al. 2021). *Mammillaria haageana* and *M. albilanata* represent a complex that extends widely in southern Mexico. Our results show that the infrageneric units of *M. haageana* and *M. albilanata* can be considered independent evolutionary units. It is possible that the variation in these inhabited environments promotes divergence in these taxa, although more in-depth studies are needed to understand and corroborate the hypotheses raised here.

The chloroplast marker sequences that we used (*rpl*16 and *psb*A-*trn*H) were not sufficient to establish the relationships among the taxa within M. ser. Supertextae. This was not surprising, as chloroplast markers have been used to resolve relationships at the species level; however, they have limitations when the species are closely related (Yan et al. 2018). This is because recently diversified groups may generate complicated genetic patterns, such as incomplete lineage sorting and hybridizations and/or introgressions (Li et al. 2016; Goetze et al. 2017), which may be true for M. ser. Supertextae. In other taxa (e.g., *Petalidium* Nees, Acanthaceae; Tripp et al. 2017), these problems have been addressed using multiple-locus methods to infer genetic trees, although they require nuclear markers that are not linked with levels of sequence variation according to phylogenetic questions (Eaton and Ree 2013). To date, no effective nuclear markers have been developed for Cactaceae, and existing markers provide fewer informative sites than chloroplast markers (Cruz et al. 2016). Recently, proposals for nuclear markers have been generated through mining strategies to test hybridization in *Opuntia* species (Granados-Aguilar et al. 2020). Currently, several methodologies have been designed that allow biological questions to be answered using a reduced representation of the genome (Anderson et al. 2017; Choquet et al. 2019; David et al. 2019). This confers advantages when working with nonmodel species such as M. ser. Supertextae, since genomic markers can be genotyped in many individuals at low cost, and in most cases, it is not necessary to have a priori information such as a reference genome (da Fonseca et al. 2016).

The taxonomic proposals of M. ser. Supertextae species have been mainly based on interpretations according to the author’s experience (Table 1), and their relationships have not been specifically tested under phylogenetic methods. Our methods are intended to be systematic (explicit criteria) and reproducible. Under this scheme, the results obtained show that within *M. haageana* and *M. albilanata*, there are genetic differences possibly indicating that these species comprise more than one taxonomic entity. Nevertheless, when distinguishing between *M. haageana* and *M. albilanata*, the task becomes difficult because both share similar distributions and habitats (mainly in southern Mexico; Fig. 2), and the morphological differences have not been well defined (Arias et al. 2012). A possible hypothesis is that there could still be gene flow between these taxa, and we might be witnessing an ongoing speciation process.

## Conclusion

By including most of the species recognized by Hunt et al. (2006), our results show that M. ser. Supertextae is monophyletic, and we corroborate that *M. eriacantha* is part of the series as previously proposed. We find that M. ser. Polyacanthae is the sister series, as proposed by (Hunt 2011). The results also showed that M. ser. Supertextae is a recently diverged group.

This is a first approximation to understand the evolutionary processes within M. ser. Supertextae. Future work should test sequencing techniques that allow genomic markers to be genotyped in many individuals since it is possible that conflicts in the phylogeny were the result of reticulate evolution. Furthermore, disentangling this problem will require a comprehensive pool of approaches regarding morphology and ecology, opening an avenue to develop M. ser. Supertextae as a model for studying complex evolutionary processes in *Mammillaria*.

## Appendix 1

List of GenBank accession numbers and vouchers for newly published sequences for all sequences used in the analyses. Data are arranged in the following order: taxon name in bold (in alphabetical order); voucher data (country, estate, locality, collecting date, collector, collecting number, *rpl*16, *psb*A-*trn*H).

***Coryphantha durangensis*** Britton & Rose, HM041405, AY545338; ***C. hesteri*** Y. Wright, AY545234, AY545342; ***C. pallida*** Britton & Rose, AY545232, AY545340; ***C. vivipara*** Britton & Rose, KC196809, KC196847; ***Escobaria chihuahuensis*** Britton & Rose, AY545233, AY545341; ***E. zilziana*** (Boed.) Backeb., AY545236, AY545344; ***Ferocactus haematacanthus*** (Salm-Dyck) Borg ex Backeb., HM041431, MH129870; ***F. latispinus*** Britton & Rose, HM041432, MH129871; ***Mammillaria albicans*** A. Berger, AY545238, AY545346; ***Mammillaria albilanata*** subsp. ***albilanata*** Backeb., México, Guerrero, 10 km east of Huitzuco, 31 October 2018, Cristian Cervantes, CC047, MT995687, MT995715; ***Mammillaria albilanata*** subsp. ***oaxacana*** D. R. Hunt, México, Oaxaca, Santiago Huauclilla-Santa Catarina Tlaxila road near the river, 1 October 2017, Cristian Cervantes, CC036, MT995698, MT995726; México, Oaxaca, 1.1 km from the junction of highway 135 Teotitlán de Flores Magón-San Fransisco Telixtlahuaca heading to San Sebastián Sedas, 2 October 2017, Cristian Cervantes, CC040, MT995689, MT995717; México, Oaxaca, Santa Maria Jalapa de Marquéz towards the microwave antenna, 22 June 2018, Cristian Cervantes, CC044, MT995678, MT995706; México, Oaxaca, 3.8 Km from Santo Domingo Tonalá to San Agustín Atenango, 24 June 2018, Cristian Cervantes, CC046, MT995677, MT995705; ***Mammillaria albilanata*** subsp. ***reppenhagenii*** (D. R. Hunt) D. R. Hunt, México, Jalisco, Tolimán, -, -, Hilda Arreola, s.n., MT995702, MT995730; ***Mammillaria albilanata*** subsp. ***tegelbergiana*** (H. E. Gates ex G. E. Linds.) D. R. Hunt, México, Chiapas, Comitán de Dominguez – 3.1 km from Comitán, 19 January 2007, Salvador Arias, SA1630, -, -; ***M. armillata*** K. Brandegee, AY545240, AY545349; ***M. bachmannii*** Boed., AY545241, AY545350; ***M. backebergiana*** Franc. G. Buchenau, AY545242, AY545351; ***M. barbata*** Engelm, AY545243, AY545352; ***M. beneckei*** Ehrenb., AY545244, AY545353; ***M. blossfeldiana*** Boed., AY545245, AY545354; ***M. bombycina*** Quehl & Quehl, AY545246, AY545356; ***M. boolii*** G. E. Linds., AY545247, AY545357; ***M. brachytrichion*** Lüthy, AY545248, AY545358; ***M. cadereytensis*** R. T. Craig, AY545249, AY545359; ***M. candida*** Scheidw., AY545250, AY545360; ***M. carnea*** Zucc. ex Pfeiff., HM041449, AY545363; ***M. cerralboa*** Orcutt, AY545254, AY545364; ***M. columbiana*** subsp. ***columbiana*** Salm-Dyck, Venezuela, Mérida, -, 2012, Teresa Terrazas, TT957, -, -; ***M. columbiana*** subsp. ***yucatanensis*** (Britton & Rose) D. R. Hunt, México, Yucatán, Municipio Dzemul, 2004, CICY, G. Carnevali & I. M. Ramírez, 7449, MT995701, MT995729; ***M.*** subsp. ***crucigera*** Mart., México, Oaxaca, 2 km from San Antonio Nahuahautipan, 9 August 1990, Ulises Guzmán, UG787, MT995697, MT995725; ***M. crucigera*** subsp. ***tlalocii*** (Repp.) D. R. Hunt, México, Oaxaca, 8 km from the rural road Santa María Tecomavaca-Santa María Ixcatlán, 9 November 1994, Ulises Guzmán, UG1103, MT995696, MT995724; ***M. decipiens*** Scheidw., AY545255, AY545369; ***M. dixanthocentron*** Backeb. ex Mottram, México, Oaxaca, km 107 highway 135 between Tecomavaca and Cuicatlán, 24 Octuber 1990, Ulises Guzmán, UG828, MT995679, MT995707; ***M. elongate*** DC., AY545258, AY545373; ***M. eriacantha*** Link & Otto ex Pfeiff., México, Veracruz, km 305 of the Xalapa-Veracruz highway between Plan del Río and Cerro Gordo, 21 January 2012, Salvador Arias, SA2169, MT995700, MT995728; ***M. flavicentra*** Backeb. ex Mottram, México, Oaxaca, Teotitlan de Flores Magón 10 Km on the way to Huautla, 13 July 1995, Patricia Novoa, CPNL132, MT995688, MT995716; ***M. formosa*** Scheidw., AY545259, AY545376; ***M. fraileana*** (Britton & Rose) Boed., AY545260, AY545377; ***M. gasseriana*** Boed., AY545261, AY545378; ***M. geminispina*** DC., AY545262, AY545379; ***M. glassii*** R. A. Foster, AY545263, AY545380; ***M. grusonii*** Runge, AY545266, AY545383; ***M. guelzowiana*** Werderm., AY545267, AY545384; ***M. haageana*** Pfeiff., México, Puebla, 1 km over the gap from the junction with the Puebla-Xalapa highway, 15 May 2017, Cristian Cervantes, CC020, MT995686, MT995714; México, Veracruz, 7 km from Perote, 15 May 2017, Cristian Cervantes, CC021, MT995693, MT995721; México, Puebla, 1.5 km south of Esperanza, 16 May 2017, Cristian Cervantes, CC022, MT995692, MT995720; México, Puebla, 7 km west of Tehuacan, 16 May 2017, Cristian Cervantes, CC023, MT995675, MT995703; México, Puebla, near the Helia Bravo Botanical Garden, 16 May 2017, Cristian Cervantes, CC024, MT995680, MT995708; México, Puebla, 9.5 km from junction 125 to Huajolotitlán towards Los Reyes Metzontla, 16 May 2017, Cristian Cervantes, CC025, MT995676, MT995704; México, Oaxaca, km 59.5 Highway 125 then join the road to San Sebastián Frontera, 17 May 2017, Cristian Cervantes, CC027, MT995683, MT995711; México, Veracruz, In La Organera area near the Tecamalucan town, 29 September 2017, Cristian Cervantes, CC029, MT995685, MT995713; México, Oaxaca, 26 Km on the Huauclilla-El Parian dirt road, 1 Octuber 2017, Cristian Cervantes, CC035, MT995690, MT995718; México, Oaxaca, 11.5 km from Magdalena Jaltepec heading to Santiago Tilangongo, 1 Octuber 2017, Cristian Cervantes, CC039, MT995691, MT995719; México, Oaxaca, 7.5 km from Corral de Piedra to Santa Maria Tutla, 22 June 2018, Cristian Cervantes, CC045, MT995684, MT995712; México, CDMX, in the Reserva Ecológica del Pedregal de San Ángel (REPSA), 6 December 2018, -, -, MT995681, MT995709; ***M. halei*** K. Brandegee, AY545269, AY545386; ***M. hernandezii*** Glass & R. A. Foster, AY545270, AY545387; ***M. herrerae*** Werderm., AY545271, AY545388; ***M. huitzilopochtli*** subsp. ***huitzilopochtli*** D. R. Hunt, México, Oaxaca, 7 km northwest of San Juan Bautista Cuicatlán, 5 August 1990, Salvador Arias, SA856, MT995694, MT995722; ***M. huitzilopochtli*** subsp. ***niduliformis*** (A.B.Lau) Pilbeam, México, Oaxaca, Río Santo Domingo up the junction Rio Salado, 12 March 1983, A. B. Lau, ABL1495, MT995695, MT995723; ***M. humboldtii*** Ehrenb., AY545273, AY545390; ***M. insularis*** H. E. Gates ex Shurly, AY545275, AY545392; ***M. jaliscana*** Boed., AY545276, AY545393; ***M. karwinskiana*** Mart., AY545277, AY545394; ***M. klissingiana*** Boed., AY545278, AY545395; ***M. lanata*** Orcutt, México, Puebla, Rio Hondo cerca del puente Calapa autopista Tehuacán-Oaxaca, 19 November 1994, Patricia Novoa, CPNL122, MT995699, MT995727; ***M. lasiacantha*** Engelm., AY545279, AY545396; ***M. lindsayi*** R. T. Craig, AY545280, AY545398; ***M. longimamma*** DC., AY545281, AY545399; ***M. luethyi*** G. S. Hinton, AY545282, AY545400; ***M. magnifica*** Franc. G. Buchenau, AY545283, AY545401; ***M. magnimamma*** Haw., AY545284, AY545402; ***M. mainiae*** K. Brandegee, AY545285, AY545403; ***M. mammillaris*** H. Karst., AY545286, AY545404; ***M. mazatlanensis*** K. Schum., AY545287, AY545407; ***M. melanocentra*** subsp. ***rubrograndis*** (Repp. & A. B. Lau) D. R. Hunt, AY545288, AY545408; ***M. mercadensis*** Patoni, AY545289, AY545410; ***M. microhelia*** Werderm., AY545291, AY545411; ***M. moelleriana*** Boed., AY545292, AY545412; ***M. mystax*** Mart., AY545294, AY545414; ***M. nazasensis*** (Glass & R. A. Foster) Repp., AY545295, AY545416; ***M. neopalmeri*** R. T. Craig, AY545296, AY545417; ***M. oteroi*** Glass & R. A. Foster, AY545297, AY545418; ***M. parkinsonii*** Ehrenb., AY545298, AY545419; ***M. patonii*** Werderm. in Backeb., AY545299, AY545420; ***M. pectinifera*** F. A. C. Weber, AY545300, AY545421; ***M. peninsularis*** Orcutt, AY545301, AY545422; ***M. pennispinosa*** Krainz, AY545302, AY545423; ***M. perezdelarosae*** Bravo & Scheinvar, AY545303, AY545424; ***M. phitauiana*** Werderm. in Backeb., AY545305, AY545426; ***M. picta*** Meinsh., HM041452, AY545427; ***M. plumosa*** F. A. C. Weber in Bois, AY545307, AY545428; ***M. polyedra*** Mart., AY545308, AY545429; ***M. pondii*** Greene, HM041399, AY545431; ***M. poselgeri*** Hildm., HM041400, AY545432; ***M. pottsii*** Scheer ex Salm-Dyck, AY545312, AY545433; ***M. prolifera*** (Mill.) Haw., AY545313, AY545434; ***M. rekoi*** Vaupel, AY545314, AY545435; ***M. rettigiana*** Boed., AY545315, AY545436; ***M. rhodantha*** Link & Otto, AY545316, AY545437; ***M. schumannii*** Hildm., AY545317, AY545438; ***M. senilis*** Lodd. ex Salm-Dyck, AY545318, AY545440; ***M. sinistrohamata*** Boed., AY545319, AY545441; ***M. sphacelate*** Mart., AY545320, AY545442; ***M. spinosissima*** Lem., AY545321, AY545443; ***M. stella-de-tacubaya*** Heese, AY545322, AY545444; ***M. supertexta*** Mart. ex Pfeiff., México, Oaxaca, 0.5 km east of San Juan de los Cues, 9 August 1990, Ulises Guzmán, UG793, MT995682, MT995710; ***M. tetrancistra*** Engelm., KC196805, KC196840; ***M. thornberi*** subsp. ***thornberi*** Orcutt, AY545324, AY545447; ***M. thornberi*** subsp. ***yaquensis*** (R. T. Craig) D. R. Hunt, AY545325, AY545448; ***M. vetula*** subsp. ***gracilis*** (Pfeiff.) D. R. Hunt, AY545327, AY545449; ***M. voburnensis*** subsp. ***voburnensis*** Scheer, AY545328, AY545450; ***M. voburnensis*** subsp. ***eichlamii*** (Quehl) D. R. Hunt, AY545329, AY545451; ***M. weingartiana*** Boed., AY545330, AY545452; ***M. wrightii*** Engelm., AY545331, AY545454; ***M. zacatecasensis*** Shurly, AY545332, AY545455; ***M. zephyranthoides*** Scheidw., AY545333, AY545457; ***Neolloydia conoidea*** Britton & Rose, HM041462, AY545458; ***Ortegocactus macdougallii*** Alexander, HM041484, AY545459; ***Pelecyphora aselliformis*** C. Ehrenb., AY545336, AY545460; ***Stenocactus lloydii*** A. Berger, AY545337, AY545461.
